# Formation of the β-barrel assembly machinery complex in lipid bilayers as seen by solid-state NMR

**DOI:** 10.1038/s41467-018-06466-w

**Published:** 2018-10-08

**Authors:** Cecilia Pinto, Deni Mance, Tessa Sinnige, Mark Daniëls, Markus Weingarth, Marc Baldus

**Affiliations:** 1 NMR Spectroscopy, Bijvoet Center for Biomolecular Research, Utrecht University, Padualaan 8, 3584 CH Utrecht, The Netherlands; 2 Present Address: Department of Chemistry and Applied Biosciences, ETH Zürich, Vladimir Prelog Weg 1-5, CH-8093 Zürich, Switzerland; 3 Present Address: Department of Chemistry, University of Cambridge, Cambridge, CB2 1EW UK

## Abstract

The β-barrel assembly machinery (BAM) is a pentameric complex (BamA–E), which catalyzes the essential process of β-barrel protein insertion into the outer membrane of *E. coli*. Thus far, a detailed understanding of the insertion mechanism has been elusive but recent results suggest that local protein motion, in addition to the surrounding membrane environment, may be of critical relevance. We have devised a high-sensitivity solid-state NMR approach to directly probe protein motion and the structural changes associated with BAM complex assembly in lipid bilayers. Our results reveal how essential BamA domains, such as the interface formed by the polypeptide transport associated domains P4 and P5 become stabilized after complex formation and suggest that BamA β-barrel opening and P5 reorientation is directly related to complex formation in membranes. Both the lateral gate, as well as P5, exhibit local dynamics, a property that could play an integral role in substrate recognition and insertion.

## Abstract (web summary)

The β-barrel assembly machinery (BAM) catalyzes β-barrel protein insertion into the outer membrane of E.coli. Here authors employ high-sensitivity solid-state NMR to reveal how the lipid environment and formation of the BamA-BamCDE complex affect BamA structure and dynamics with regards to the lateral gate and the β-barrel associated domains.

## Introduction

Membrane protein function is closely influenced by the presence of a surrounding lipid bilayer^1,2^ and the ability to dynamically switch between different functional states^3,4^. Both aspects are known to control processes such as signal transduction^5,6^ or molecular transport^7,8^ and recent evidence implicates these phenomena in the insertion and folding of outer membrane proteins via the β-barrel assembly machinery (BAM) of *E.coli*^9,10^.

The BAM protein machinery is essential for physiological, pathogenic, and drug resistance functions^11^ and consists of the central component BamA and four accessory lipoproteins (BamB, BamC, BamD, and BamE). The polypeptide transport associated domains (POTRA domains) of BamA serve as a central hub for lipoprotein organization^12^. In particular, POTRA 5 (P5) contains the crucial interface between the only two essential proteins of the complex^13,14^, BamA that forms a β-barrel and BamD that adopts a 5 tetratricopeptide fold. X-ray and cryo-electron microscopy (cryo-EM) structures^15–18^ have also revealed the assembly of the complex and its remarkable structural variability in micelles of various detergents^10,19^.

In spite of these advancements, a detailed understanding of how the BAM complex carries out its function without requiring ATP, that is critical in Sec translocon-mediated protein insertion in the inner membrane^20^, has remained under debate^10,21^. Local perturbation of the bilayer, lipoprotein interactions, lateral pressure exerted by the surrounding asymmetric environment, as well as the nascent β-barrel itself are all likely to play an important role in modulating the function of the complex^22–25^. In addition, recent work suggests that key protein regions of BamA, in particular the previously identified lateral gate between β-strand 1 and β-strand 16^15–18^, as well as the widely conserved β-strands of P5 are critically involved in unfolded OMP insertion^12,15^. In this process, the lateral gate may actually fulfill two functions. Firstly, it would be primarily involved in folding of substrates by dynamic interactions between BamA β-strands 1 and 16 and the substrate protein via a process called β-augmentation, as recently discussed for Sam50, a close homolog of BamA in mitochondria^26^. Secondly, the lateral gate would play a key role for release of substrates into the bilayer, such as seen in the SecYEG system that inserts helical proteins into the inner membrane^27^ by local destabilization of the membrane bilayer^19^. Notably, such a bilayer state of BamA has thus far remained elusive to X-ray and cryo-EM studies.

Solid-state NMR (ssNMR) provides a spectroscopic tool to infer membrane protein structure and dynamics in a lipid bilayer^28–31^ and, more recently, in the native membrane environment^32,33^. Applied to BamA we previously showed^34–36^ that the BamA β-barrel can accommodate membrane bilayers of varying hydrophobic thicknesses and that the POTRA domains do not display fast global motion in proteoliposomes^34^.

In the following, we examine how the lipid environment and formation of the BamA–BamCDE complex affect BamA structure and dynamics with regards to the lateral gate, the P4P5 domains and extracellular loop 6 (EL6), which are all critically involved in function^37^. To tackle the spectroscopic challenges related to dealing with large membrane protein (complexes), we combine recent advancements in high-sensitivity ssNMR, i.e., dynamic nuclear polarization (DNP, see e.g., refs. ^38,39^) and ^1^H-proton-detected ssNMR (see e.g., ref. ^40–42^), in conjunction with tailored ssNMR experiments and specific isotope labeling.

Our results reveal how essential BamA regions, such as the P4P5 interface become stabilized after complex formation and suggest that the BamA barrel opening is directly related to complex formation in membranes and P5 reorientation. Both the lateral gate as well as the P5-barrel interface exhibit local dynamics, a property that may be critical for substrate insertion.

## Results

### The P4P5 domains of BamA in lipid bilayers

**Fig. 1:**
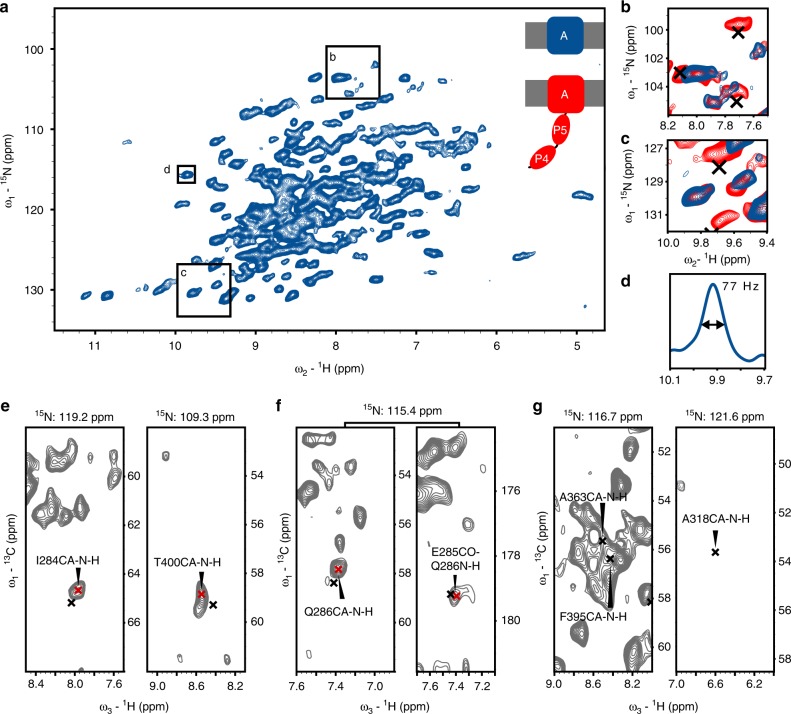
2D and 3D ^1^H ssNMR of BamAP4P5 and BamA TM in proteoliposomes. **a** ^1^H detected 2D NH spectrum of BamA TM in liposomes. Close-ups of **b** and **c** show correlations for the POTRA domains. **d** Representative ^1^H line-width extracted from **a**. **e** Representative 2D slices from a 3D CαNH experiment that show correlations for residues I284 and T400 which are within 1 ppm in all three frequencies from the solution NMR assignments. **f** Sequential assignment of the E285/Q286 pair based on solution assignments and ssNMR 3D CαNH and CONH spectra. **g** From a CαNH experiment in a region of spectral overlap, A363/F395, or e.g. A318 that does not agree with the ssNMR data. Inset in **a** is a representation of the protein constructs used. In all panels red crosses are ssNMR assignments and in black are solution NMR assignments (BMRB 19928). In **g**, the ^15^N resonance frequencies of the relevant 2D planes are indicated

**Fig. 2:**
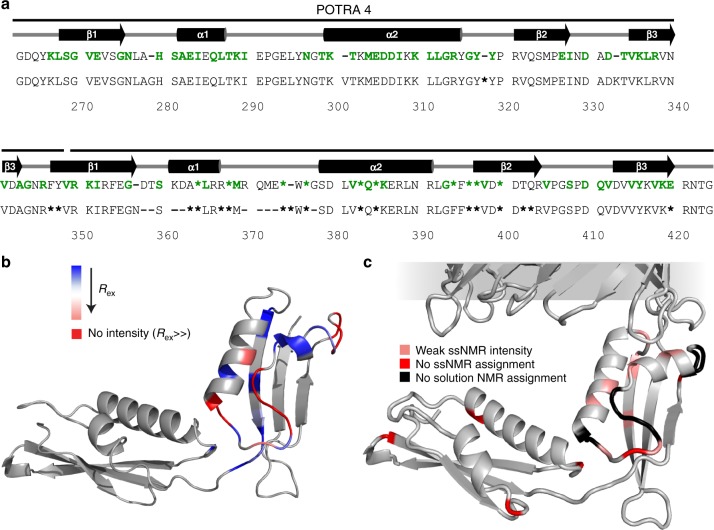
P4P5 domains in liposomes compared to free solution. **a** Topology and secondary structural elements determined using TALOS+ (BMRB 19928) depicted above the sequence for the P4P5 domains. Upper sequence: Residues identified in ^1^H-detected ssNMR spectra (green and bold), those replaced with dash (-) do not appear in the spectra within 1 ppm of their solution NMR assignment. Residues replaced with asterisks (*) show weak signals. Lower sequence: Residues replaced with asterisks (*) show dynamics as judged by ^15^N CPMG relaxation (solution NMR). Residues replaced with a dash (**-**) could not be assigned in solution. **b** R_ex_ plotted on the crystal structure of P4P5 (PDB 3Q6B). Box in the top left indicates the extent of chemical exchange. Adapted from Structure, 23, Sinnige, T. et al., Conformational Plasticity of the POTRA 5 Domain in the Outer Membrane Protein Assembly Factor BamA, 1317–1324, copyright (2015) with permission from Elsevier^35^. **c** P4P5 residues that are not present at the solution NMR assignment in liposomes, in both the CαNH and the CONH ssNMR spectra (red) plotted on the crystal structure (PDB 5D0O), in light red are residues with weak ssNMR intensity and in black are those which were not assigned in solution

**Fig. 3:**
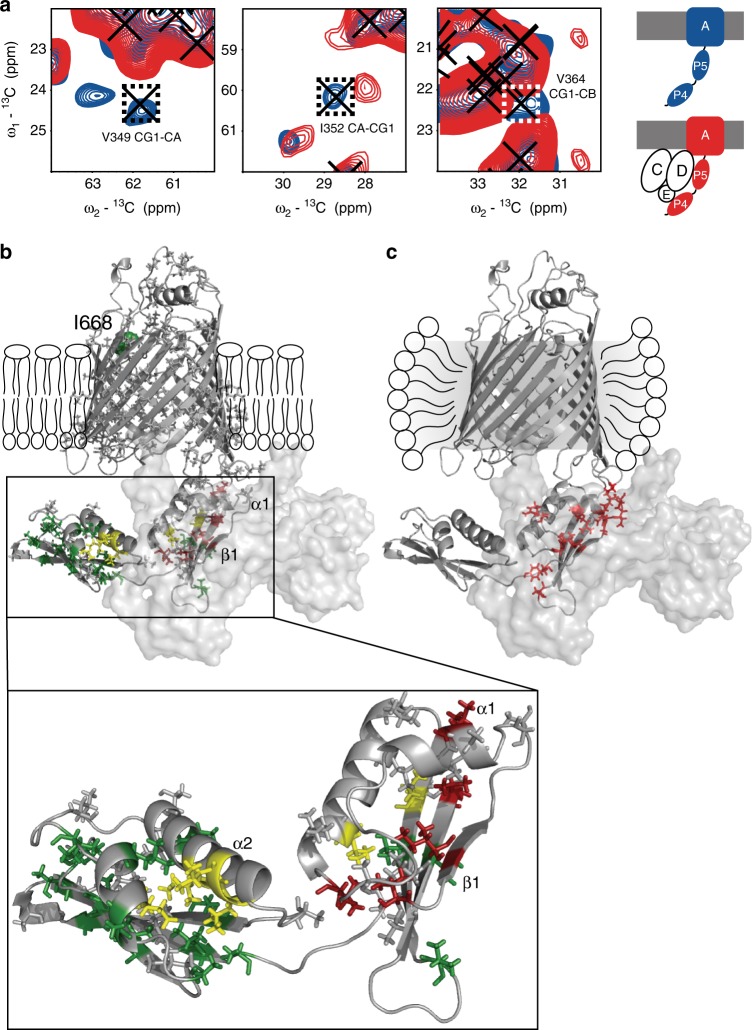
BamAP4P5–BamCDE interface in bilayers. **a** Close up of representative correlations from the 2D ^13^C–^13^C spectra of AVLTI labeled BamAP4P5 in liposomes, in the absence and presence of BamCDE (blue and red spectra, respectively). Crosses are assignments of the labeled residues; dashed boxes indicate correlations with chemical shift perturbations above 0.7 ppm. **b** BamAP4P5–BamCDE interaction interface as described by ssNMR data in lipid bilayers. Residues as sticks are labeled (i.e. AVLTI), those in gray were not used for analysis. Residues in red show a chemical shift perturbation, those that in yellow indicate an increase in intensity and those that in green do not experience any effect upon complex formation. The zoom-in shows in more detail the interaction interface. Indicated are the structural elements mentioned in the text and residue I668 of EL6 (spheres). **c** BamA–BamCDE interface as described by all BAM complex crystal structures (PDB 5D0O, 5D0Q, 5AYW, and 5EKQ). Contacts with the BamCDE complex, in red, were found using PDBePISA^29^ on these structures. BamD and BamE are shown as semi-transparent surfaces in **b**, **c**, as well as a schematic representation of the liposome bilayer and detergent micelle are present to aid visualization

To facilitate assignment of the P4P5 domains in lipid bilayers we compared three-dimensional ^1^H-detected spectra of the perdeuterated^43^ β-barrel domain of BamA (BamA TM, Fig.1a–c, blue, 387 residues) to spectra of the BamAP4P5 construct (Fig.1b, c, red, 547 residues). Proteoliposome preparations of both proteins in general exhibited narrow ^1^H line-widths in 2D (Fig. 1d) and 3D CαNH and CONH spectra. We also compared the ssNMR results to our previous solution NMR assignments (BMRB 19928)^36^ of the soluble P4P5 domains (Fig. 1b, c, e–g, black and Supplementary Fig. 1 for a global comparison). For many residues of the P4P5 domains, such as those shown for residues I284, Q286, and T400 in Fig. 1e, f, we could identify ssNMR correlations that were within 1 ppm of the solution NMR assignments for all three NMR frequencies. On the other hand, regions of spectral overlap in our 3D ^1^H-detected experiments (e.g., A363 and F395 in Fig. 1g) were not taken into account. In total, we assigned approximately 50% of the residues in P4P5 that are located throughout the secondary structural elements in both domains (Fig. 2a—upper sequence, residues in green and Supplementary Table 1.), implying that these domains retain the same overall fold as in solution and most likely have limited interaction with the BamA TM domain or the membrane.

Previously, we^35^ had identified a region of conformational plasticity in soluble P5 (Fig. 2a lower sequence and 2b, residues in light and dark red). Interestingly, many of those residues that exhibited elevated relaxation profiles in solution NMR experiments, correspond in our ssNMR data to correlations that show either low signal intensity (Fig. 2c, light red) or are absent (e.g., A318 in Fig. 1g and Fig. 2c, dark red, Supplementary Table 2). These observations confirmed our preliminary ssNMR analysis^35^ and strongly suggest that the dynamics observed for free P5 in solution are preserved in membrane-embedded BamA. Unlike in other NMR studies of membrane proteins where protein dynamics can change significantly when moving from membrane mimetics to bilayers (see. e.g., ref. ^4^), this observation may be of functional relevance. Indeed, the region of dynamics involves β2, α2 and the loop region between α1 and α2. β2 in particular has been implicated in β-augmentation^12,44^.

### BamAP4P5–BamCDE interaction

We further used ssNMR to study the interaction between BamA and BamD, the only other essential complex member^13^, in the context of the proteoliposome BamAP4P5–BamCDE sub-complex. In order to obtain residue-specific information on the formation of the complex we employed a specific ^13^C/^15^N isotope-labeling scheme to target the P5-BamCDE interaction interface, where only alanine, valine, leucine, threonine and isoleucine residues from BamAP4P5 are ^13^C/^15^N labeled (AVLTI labeling, Supplementary Fig. 2). To study the 130 kDa complex (BamAP4P5–BamCDE) we used a lipid-to-protein ratio (LPR) of 50:1 (mol/mol) or 1:4 (w/w) and 1:2 (w/w) for BamAP4P5 alone, which compares favorably to the composition of the bacterial outer membrane itself^45^. Along similar lines, we had previously shown that the BamA protein is well folded and inserted into the bilayer at a LPR of 25:1 (mol/mol)^34^ and Hussain et al. highlight how the BAM complex is functional in membranes of various compositions, including DLPC^46^.

In Fig. 3a, we compared ssNMR ^13^C–^13^C data obtained on specifically AVLTI labeled BamAP4P5 in the absence (blue) and presence (red) of the unlabeled BamCDE sub-complex in lipid bilayers (see Supplementary Fig. 3 for full spectra). For the identification of chemical-shift changes in the aliphatic spectral region, we supplemented our ssNMR backbone ^13^Cα/^13^CO ssNMR chemical shifts discussed in Fig. 2 with side-chain ^13^C resonances obtained earlier (BMRB 19928)^36^. In order to constitute a reliable indicator of change we required that at least two of the observed ssNMR correlations for a given residue deviated by more than 0.7 ppm in at least one spectral dimension when comparing BamP4P5 spectra before and after complex formation. For example, in Fig. 3a, residues V349, I352, and V364 exhibit such as a chemical-shift perturbation between the two preparations of BamAP4P5. Hence, they are likely influenced by the proximity of the BamCDE subcomplex or result from (possibly allosteric) re-orientations of the domains upon BamAP4P5–BamCDE complex formation.

Analysis of the spectra revealed a number of residues that exhibit chemical-shift changes (Fig. 3b, red) or increased signal intensities (Fig. 3b, yellow) when compared to a reference residue in the β-barrel, L630, that is rigid (Supplementary Fig. 4). In addition, we identified residues whose ssNMR signals are unaffected by complex formation (Fig. 3b, green). Residues that show chemical-shift changes in our ssNMR data cluster to β1, α1 and the adjacent random coil region of P5. This protein region is consistent with the interface seen in detergent solubilized BAM crystals as shown in Fig. 3c where residues that are directly involved in the interaction via hydrogen bonding or salt bridges are shown in red. Interestingly, the putative binding region seen in lipid bilayers partially overlaps with the dynamic region of conformational exchange identified in P5^35^. Additionally, we previously showed that with ssNMR the P4P5 interface was dynamic, causing the P4 domain to become more mobile at higher temperatures relative to the membrane-embedded β-barrel in BamA proteoliposomes^35^. Residues located at the P4P5 interface, mainly in α2 of P4 (P4: I284, I308, L311, and L312 and P5: V382 and V398, Fig. 3b yellow and corresponding zoom-in) exhibit a significant increase in ssNMR signal intensity consistent with a structural or dynamical stabilization after complex formation in spite of the lack of direct interactions between P4 and the BamCDE subcomplex. While formation of the complex hence leads to a reduction of inter-domain P4P5 dynamics, I668, and consequently EL6, do not experience large degrees of motional freedom after complex formation (Fig. 3b, green spheres). This observation is in accordance with previous work that shows that the EL6 loop is tethered to the interior of the transmembrane domain via the conserved VRGF motif (residues 660–664)^25^.

### Influence of complex formation upon BamAP4P5 conformation

**Fig. 4:**
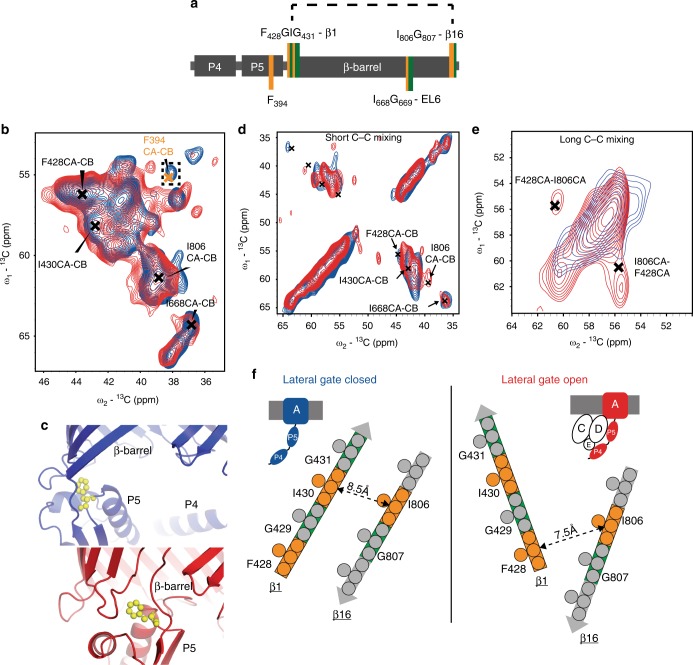
Influence of BamCDE on the BamAP4P5 lateral gate and P5-barrel orientation. **a** Localization of the sequential ^13^C Ile, Phe (orange) and ^15^N Glycine (green) pairs targeted in **d**–**f** For completion, also F394 discussed in **b**, **c** is shown. **b** 2D ^13^C–^13^C spectra of the specifically ^13^C Ile, Phe labeled BamAP4P5 in liposomes, in the absence (blue) and presence (red) of sub-complex BamCDE. F394 at the P5-barrel interface is indicated in yellow. **c** Comparison of the P5-barrel orientation as described by X-ray structures (PDB 5D0O—blue and 5D0Q—red) with F394 in yellow. **d**, **e** Close-up of the Cα-Cβ region of the ^15^N-edited C_*x*_C_*x*_ DNP enhanced spectra with a short (30 ms—**d**) or long (1 s—**e**) mixing time, measured on IFG-labeled BamAP4P5, in the presence and absence of unlabeled BamCDE (blue and red spectra, respectively). Crosses are the tentative assignments for the residues targeted by these experiments. **f** Schematic representation of the correlations observed between β-strand 1 and 16 in ^15^N-edited C_*x*_C_*x*_ DNP spectra for short and long mixing times (arrows in full and dashed, respectively) for the case of a lateral gate closed (blue) and open (red). The distances between Cα residues of the closest residue in β1 to the I806 of β16 are indicated, whilst other inter-strand distant are significantly greater, as judged from PDBs 5D0O and 5D0Q, respectively

Next, we devised an ssNMR approach utilizing specific labeling of ^13^C isoleucine and phenylalanine and ^15^N glycine (^13^C–Ile, ^13^C–Phe and ^15^N–Gly labeling) to probe the P5-barrel interface as well as the lateral gate and EL6 after lipoprotein binding in DLPC bilayers. This strategy allowed us to monitor P4P5 Ile and Phe residues for which assignments are available and created four unique sequential pairs across the entire BamA construct, in particular within the lateral gate strands 1 and 16 as well as in EL6 (Fig. 4a, Supplementary Fig. 5a for sequence).

Analysis of ^13^C–^13^C correlation spectra before (blue) and after (red) complex formation (cutout in Fig. 4b and full spectra in Supplementary Fig. 6a) showed that while overall the spectra are comparable, several residues exhibit chemical-shift perturbations. Of these we could identify F394 from P5 (Fig. 4b orange), located at the P5-barrel interface. This change in local environment is in line with the movement of P5 to beneath the TM domain as seen in the X-ray structures (Fig. 4c residue in orange).

To unambiguously detect changes in the lateral gate and EL6 in the Bam complex, we combined conventional and DNP ssNMR to probe sequential specifically labeled pairs (Fig. 4a). DNP greatly increased the signal to noise ratio which, compared to our ^1^H studies, is compromised by the increased protein size (130 kDa vs. 57 kDa) and the need to invoke rare spin (^15^N–^13^C and ^13^C–^13^C) polarization transfer steps to select specific sequential correlations in our ssNMR spectra. In the current context, we observed DNP enhancements factors of about 110 using a 400 MHz/263 GHz DNP system.

In order to obtain assignments for the sequentials of interest we combined information from the ^13^C–^13^C correlation spectrum (Fig. 4b, blue) with results of a 1D ^15^N-edited C_O_C_*x*_ and correlations found in 2D NCO experiments^47^ recorded at an effective sample temperature of approximately −2 °C (Supplementary Fig 5b). These data were subsequently supplemented with ^15^N-edited C_*x*_C_*x*_ experiments^48^ recorded under DNP conditions that only contain signals from sequential ^13^C–^15^N labeled residue pairs (Fig. 4d, e). With this strategy we were able to tentatively assign four residues, namely F428, I430 and I806 of β1 and β16, in addition to the helical I668 residue of EL6^21^ (Fig. 4d, e, black crosses, see also Supplementary Fig 5b and Supplementary Table 3).

To investigate β1 and β16 and their relative distance we compared DNP-supported ^15^N-edited C_*x*_C_*x*_ experiments for short and long mixing times (see Supplementary Fig. 7 for diagram of the experiments and Supplementary Figures 6b and c for entire spectra). As expected, the resulting spectra before (Fig. 4d, blue) and after (Fig. 4d, red) complex formation using short mixing times were similar with the exception of I806 that strongly increased in signal intensity upon addition of the BamCDE sub-complex (Supplementary Fig. 6d). This aspect could stem from structural disorder at low temperature that is not observed at ambient temperature (where we could assign the Cα, Cβ and CO of I806).

To probe the conformation of the lateral gate, we applied a longer mixing time for the ^15^N-edited C_*x*_C_*x*_ experiment (Fig. 4e). This allows for magnetization transfer to occur between residues beyond the sequential pairs and between close-by residues of β-strands 1 and 16. According to the X-ray results (Fig. 4f), gate opening leads to a relative movement of the two strands that affects the inter-atomic distances of the spin pairs probed in our ssNMR experiments. For the case of an open lateral gate (Fig. 4f, right panel), F428 is nearer to I806. Indeed, we identified a correlation that clearly stems from the F428–I806 residues in the sample of BamAP4P5 in complex with BamCDE indicating their proximity in this complex (Fig. 4e, red). Note that such a correlation was not observed before formation of the complex (Fig. 4e, blue and Supplementary Fig. 5e and f for the comparison of peak intensities). For the case in which the lateral gate is closed (Fig. 4f, left panel) we were not able to observe polarization transfer between I430 and I806. As mentioned above, since we also did not observe intra-residue correlations for I806 for short mixing times, in the absence of complex formation (Fig. 4d), we attribute these findings to structural disorder of residue I806 at low temperatures. Taken together our data hence suggest that upon binding of the lipoproteins BamCDE, the conformation of P4P5 is altered in regard to the β-barrel, and the lateral gate is open in our lipid environment.

## Discussion

Considerable progress has been made in obtaining structural insight into the assembly of the BAM complex. Yet, X-ray and cryo-EM structures also revealed that the BAM complex can adopt multiple conformations in membrane mimetics. Furthermore, computational studies that have tried to reconcile overall and local motion in the BAM machinery in the context of BAM function have led to conflicting results^25,49^. Here we have shown how the combination of high-sensitivity ssNMR methods and specific isotope labeling can be used to track protein structure and motion before and after formation of the BAM complex in lipid bilayers. Previous work has shown that bilayer preparations of various compositions, including DLPC^46^ are functional^18,50^ and lead to substrate insertion. Moreover, protein reconstitution levels utilized in our study compare favorably to protein concentrations of the bacterial outer membrane itself^45^.

**Fig. 5:**
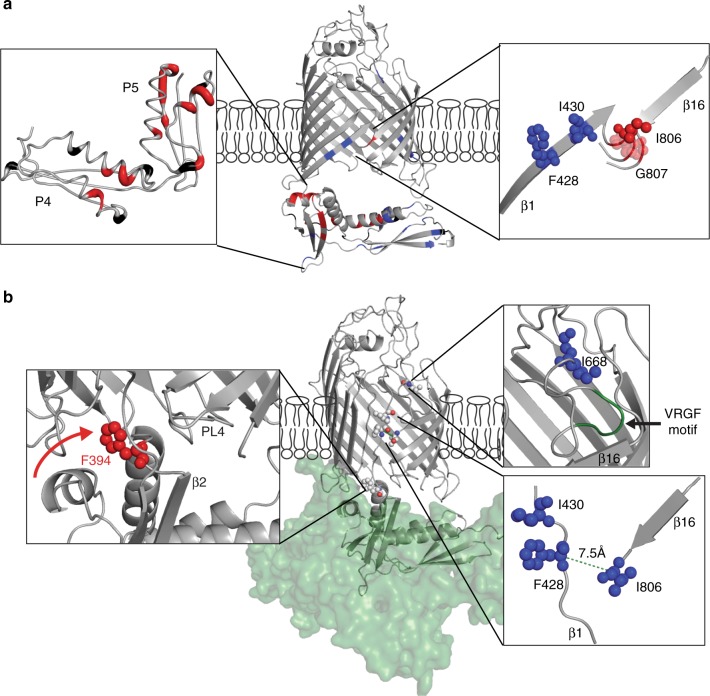
Structure and dynamics of BamAP4P5 are influenced by complex formation. **a** Dynamic BamAP4P5 protein region (indicated in red) as judged from ssNMR (^1^H-detected and DNP) experiments in proteoliposomes. In blue are residues which show no significant changes upon complex formation. In black are residues that could not be assigned by ssNMR CαNH and CONH experiments. **b** Conformational changes within BamAP4P5 upon complex formation with the sub-complex BamCDE. Inset at left is the movement of P5 to beneath the β-barrel, indicated by the red arrow, a movement that is anchored in place by residues from periplasmic loop 4 and residue F394 (red), which exhibits chemical shift perturbation in ssNMR experiments. Inset at right top, the EL6 loop anchored to the barrel via the conserved VRGF motif, which exhibits no changes as judged by the proximal I668. Inset right bottom: distance measurement between I806 and F428 that allows to follow lateral gate opening occurring upon complex formation

Firstly, we detect significant protein dynamics before complex formation at both the lateral gate as well as in the interface region of P5 and the β-barrel (Fig. 5a) which has previously been shown to be critical for BAM function^37^. Interestingly, previous X-ray structures observed a kink in β16^37^ in free *E. Coli* BamA in detergents. This kink is only one residue away from I806, which exhibits structural disorder at ssNMR DNP conditions before complex formation (Fig. 5a). This difference to our bilayer studies may point to an active role of the surrounding bilayer in facilitating local structural flexibility (but not gate opening) in the resting state of BamA at the lateral gate. Similar to the recent case of Sam50, such local flexibility at the gate and within the P5-barrel interface could be critical for substrate recognition and folding via β-augmentation. In this notion, the substrate would transiently bind to the β2 strand of P5 which is, in the case of the BamAP4P5–BamCDE complex (Fig. 5b left panel), positioned favorably to allow direct access to the open lateral gate.

Secondly, our data suggest that complex formation with BamCDE stabilizes membrane embedded BamA and drives reorientation of both P4 and P5 domains, a motion that triggers lateral gate opening. The BamA–BamCDE sub-complex interface and the structural changes between the lateral gate β-strands 1 and 16 seen by our ssNMR studies in membranes (Fig. 5b) are similar to what is observed in detergent solubilized complexes from X-ray and cryo-EM.

Thirdly, our results indicate that the interaction of the subcomplex BamCDE with BamA occurs independently of the P1P3 domains and that the P4P5 segment is necessary and sufficient to interact with BamCDE. These findings are in line with the earlier work^51–53^ showing that deletions of other lipoproteins are tolerated for BAM function except for BamD, that is essential in orienting the POTRAs correctly below the β-barrel and consequently driving β-barrel opening. In fact, the previous work has shown that to ensure efficient OMP insertion, the POTRA and β-barrel domains must be precisely aligned^54^ and suggests that the other lipoproteins aid in alternating the complex between different conformations^55,56^.

Taken together, our work connects previous structural studies to the presence of local protein dynamics that are closely related to the formation of the BAM complex in lipid bilayers and that may be critical for BAM function without external sources of energy Whereas the SecYEG translocon requires the association with either a translating ribosome (in co-translational translocation) or with the SecA ATPase (in post-translational translocation) to drive insertion or translocation, the BAM complex, and mainly the core component BamA and only other essential lipoprotein BamD are necessary and sufficient to assemble an active conformation that could promote folding. We propose that this process occurs mainly due to the existence of multiple points of dynamics within the BamA protein (Fig. 5a) that when the complex is formed leads to conformational changes (Fig. 5b) that are conducive to substrate folding. In this scenario, the additional lipoprotein BamB and/or complete insertion of the OMP would lead to conformational cycling back to the resting lateral gate closed state. Reminiscent of emerging signal-transduction mechanisms across cell membranes^6,57^, the modulation of protein structure as well as global and local dynamics may hence be sufficient to shift the conformational equilibrium from inactive to active protein states.

## Methods

### Protein production and ssNMR sample preparation

BamAP4P5 (D264–W810) and BamA TM (G424–W810) were prepared as described previously^34^. Briefly, for ^1^H ssNMR experiments, the samples (BamAP4P5 and BamA TM) were produced in *E.coli* BL21 (DE3) Star (Invitrogen) as cytoplasmic inclusion bodies. These cells were grown in a perdeuterated (U–^2^H, ^13^C, ^15^N) M9 minimal medium (100% D_2_O) containing 100 μg ml^−1^ ampicillin. Protein expression was achieved by growing the cells to an OD_600_ of 0.6–0.8 prior to induction with 1 mM IPTG, after which the cells were harvested after 4 h at 37 °C. Inclusion bodies of these proteins were purified^22^ and solubilized in a buffer containing 20 mM Tris-HCl, pH 8.0, 100 mM glycine and 6 M urea. The proteins were refolded by rapid 10-fold dilution into a buffer containing 50 mM sodium phosphate, pH 7, and 1% *N*,*N*-dimethyl-*N*-dodecylamine-*N*-oxide (LDAO, Sigma-Aldrich). Samples were further left to incubate at room temperature overnight and reconstituted into 1,2-dilauroyl-*sn*-glycero-3-phosphocholine (DLPC) lipids at a 10:1 (mol/mol) LPR by dialysis at 4 °C. The dialysis was performed against 20 mM sodium phosphate, pH 7, and 5 mM MgCl_2_ until liposomes were formed. For the specifically labeled samples, *E.coli* BL21 Star (DE3) cells (Invitrogen) transformed with the BamAP4P5 plasmid were grown in unlabeled M9 medium to an optical density of ~0.6 and induced with 0.5 mM IPTG for approximately 30 min prior to the addition of labeled and unlabeled amino acids at a final concentration of 200 mg per liter. For AVLTI labeling ^13^C, ^15^N-labeled alanine, valine, leucine, threonine and isoleucine were added and for ^13^C–Ile, ^13^C–Phe and ^15^N–Gly labeling ^13^C isoleucine and phenylalanine and ^15^N glycine were used. All other amino acids were added in unlabeled form to minimize isotope scrambling in the context of our ssNMR experiments. After purification, these specifically labeled samples were reconstituted into DLPC lipids at a 50:1 (mol:mol) LPR by dialysis at 4 °C against 20 mM sodium phosphate pH 7 until liposomes were formed.

BamAP4P5–BamCDE proteoliposome samples were prepared essentially as described in ref. ^33^, except all BamCDE preparations were unlabeled and BamAP4P5 was labeled as described above for the desired labeling scheme. For expression of the BamCDE complex, E.coli BL21 (DE3) Star (Invitrogen) cells were co-transformed with the plasmids for BamCD (pSK46) and BamE (pBamE–His)^52^. These cells were grown in unlabeled minimum medium to an OD_600_ of 0.6–0.8 prior to induction with 0.5 mM IPTG. The cell cultures were then grown for an additional 4 h at 37 °C before they were harvested. The bacterial cell pellet was resuspended in 10 mM Tris-HCl, pH 8, lysozyme and 1 μL DNase I (Sigma-Aldrich) per liter of culture. The cells were lysed by French press followed by 20 min centrifugation at 4000×*g*, 4 °C. Subsequently, the cellular membranes were harvested by ultracentrifugation at 60,000×*g* for 1 h at 4 °C (Beckman Coulter Optima L-90 K with SW32 TI rotor). These membranes were resuspended and solubilized for 2 h at 4 °C in a buffer containing 50 mM Tris-HCl pH 8, 150 mM NaCl, 1% n-Dodecyl β-D-maltoside (DDM, Sigma-Aldrich), 10 mM imidazole and protease inhibitor cocktail (Roche). The resulting supernatant was applied to Ni-NTA agarose beads (Qiagen) pre-equilibrated with 50 mM Tris-HCl, pH 8, 0.03% DDM, 10 mM imidazole and protease inhibitor cocktail (Roche) (buffer A). The Ni-NTA beads were washed with 20 column volumes of buffer A containing 25 mM imidazole followed by elution of the protein in a buffer A containing 300 mM imidazole. The sample was concentrated utilizing an Amicon ultra centrifugal filter with a 30 kDa cut-off and injected into a buffer A (without imidazole) pre-equilibrated Superdex 200 HiLoad 16/60 gel filtration column (GE).The BamAP4P5–BamCDE complex was formed by equimolar combination of the detergent solubilized proteins under mild rotation for 1 h at 4 °C and complex formation was judged by gel filtration on a HiLoad 26/600 Superdex 200 pg (GE) prior to dialysis (Supplementary Fig. 8 shows a typical curve obtained). For samples of the BamAP4P5–BamCDE complex, the complex was reconstituted into DLPC lipids at a 50:1 (mol:mol) LPR by dialysis at 4 °C against 20 mM sodium phosphate, pH 7, until liposomes were formed.

### NMR spectroscopy

For individual 2D and 3D spectra details, please refer to Supplementary Tables 4, 5 and 6 in the supplementary information.

The ^1^H-detected experiments were carried out at 18.8 T static magnetic field (800 MHz^1^ H frequency) and 58 kHz MAS with a sample temperature of 300 K. Water suppression was achieved with the MISSISSIPPI (ref. ^58^) scheme and decoupling was performed with the PISSARRO^59^ scheme during all direct and indirect acquisition periods. For all experiments and all nuclei, the decoupling amplitude was set to one quarter of the MAS frequency, i.e., 14.5 kHz.

For the 3D Cα/CONH experiments (see ref. ^42^ for pulse sequence), the initial ^1^H → ^13^C transfer was brought about with ramped (20%) cross polarization contact times of 3/3.4 ms respectively. Polarization was transferred further from ^13^Cα/CO → ^15^N with SPECIFIC CP (ref. ^60)^ using 39 kHz irradiation on ^13^C and 20 kHz irradiation on ^15^N during 4 ms. The final transfer of ^15^N → ^1^H was carried out with cross-polarization (35 kHz on ^15^N, 95 kHz on ^1^H, 750 µs contact time).

All ^13^C ssNMR experiments were performed on a Bruker AVANCE III spectrometer operating at 700 MHz ^1^H frequency (16.4 T) and equipped with a 3.2 mm ^1^H, ^13^C, ^15^N MAS probe (Bruker BioSpin). The MAS frequency was set to 13 kHz and the effective sample temperature was maintained at −2 °C. Hartman–Hahn cross-polarization (CP) was performed with a linear ramp from 70% to 100%. Contact time was optimized for each sample and typically found to be between 800 and 1000 µs. ^13^C–^13^C magnetization transfer was achieved using the PARIS pulse sequence^61^ with a 30 ms mixing time. For decoupling, SPINAL64^36^ was applied with 78 kHz irradiation on ^1^H. Spectra were identically processed using Bruker TopSpin 3.0 and analyzed in Sparky^37^. Comparison of intensities (Supplementary Fig. 4) was performed with residue L630 as control, similarly to that performed in ref. ^35^.

For DNP measurements, a solution with a concentration of 15 mM AMUPol^62^ was prepared in 20:70:10 d_8_-Glycerol:D_2_O:H_2_O containing 20 mM phosphate buffer pH 7. The protein pellet was resuspended in the radical solution. Subsequently, the sample was  centrifuged at ~45,000 rpm for 30 min to up to an hour to form the desired pellet from which the supernatant is removed. The procedure was repeated once more in order to ensure consistency before packing the sample into the rotor. The final pellet was packed into a 3.2 mm sapphire rotor prior to the measurements.

DNP measurements were conducted on a 400 MHz/263 GHz DNP system (Bruker Biospin). Samples were cooled down to 100 K and spun at 8 kHz MAS. The 2D ^15^N-edited C_*x*_C_*x*_ spectrum was recorded by using a SPECIFIC CP (ref. ^60^) ^15^N–^13^C transfers, followed by proton-driven spin diffusion (PDSD) before and after *t*_1_-evolution. 83 kHz ^1^H decoupling using SPINAL64^36^ was employed during evolution and detection periods.

## Electronic supplementary material

Supplementary Information

## Data Availability

Data supporting the findings of this manuscript are available from the corresponding author upon reasonable request.
