# Supplementary material for: Formation of the β-barrel assembly machinery complex in lipid bilayers as seen by solid-state NMR
Source: Nat Commun. 2018 Oct 8;9:4135. doi: 10.1038/s41467-018-06466-w (PMC6175958; doi:10.1038/s41467-018-06466-w)
Supplement: Supplementary file 1 — Supplementary Information [file 41467_2018_6466_MOESM1_ESM.pdf]

Supplementary Information

Formation of the β-barrel assembly machinery complex in lipid bilayers as seen by solid-state NMR

Pinto *et al.*

Supplementary Figures 1-8

Supplementary Tables 1-6

Supplementary references

Supplementary Figures

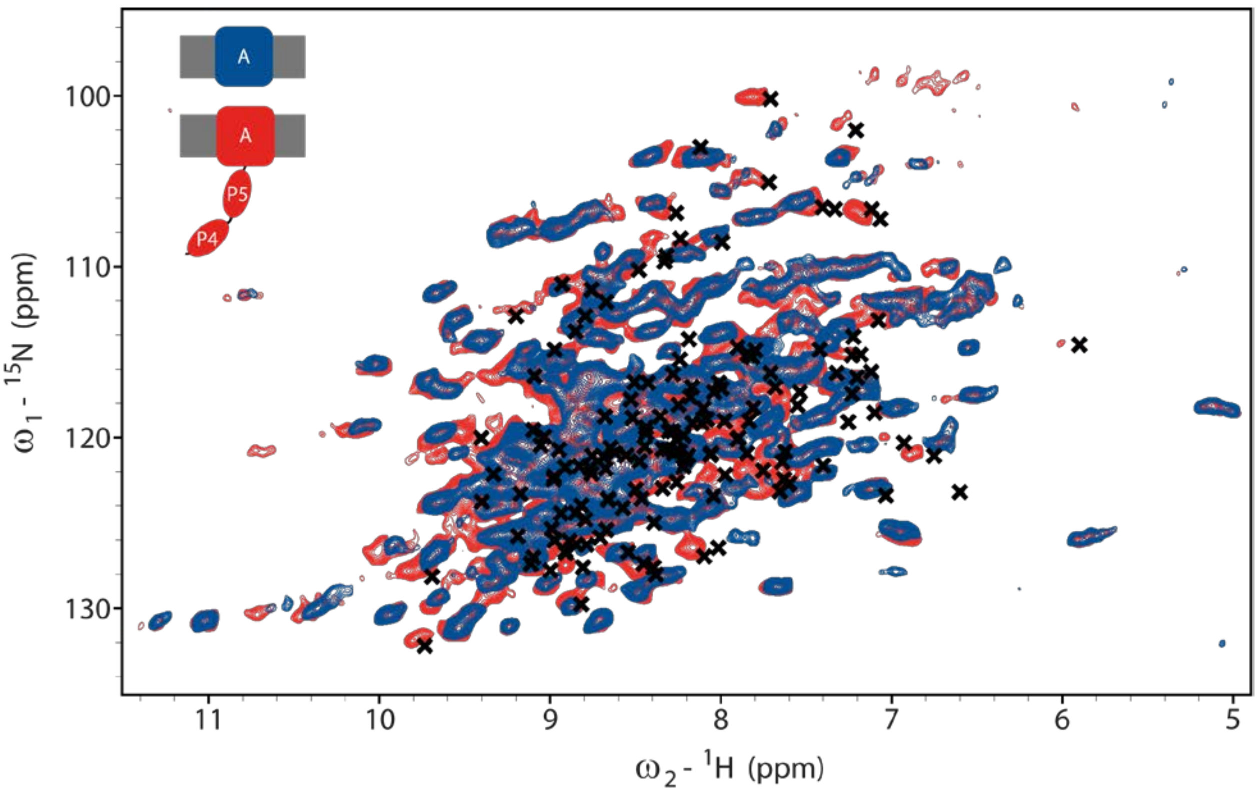

Supplementary Figure 1 Comparison of BamA TM and BamAP4P5 ^{1}H 2D spectra

Overlay of the 2D NH spectra of the BamA TM (blue) and BamAP4P5 (red) constructs recorded on an 800MHz spectrometer. Black crosses indicate the solution NMR assignments (BMRB 19928) for P4P5. Inset are the graphical representations of the constructs utilized.

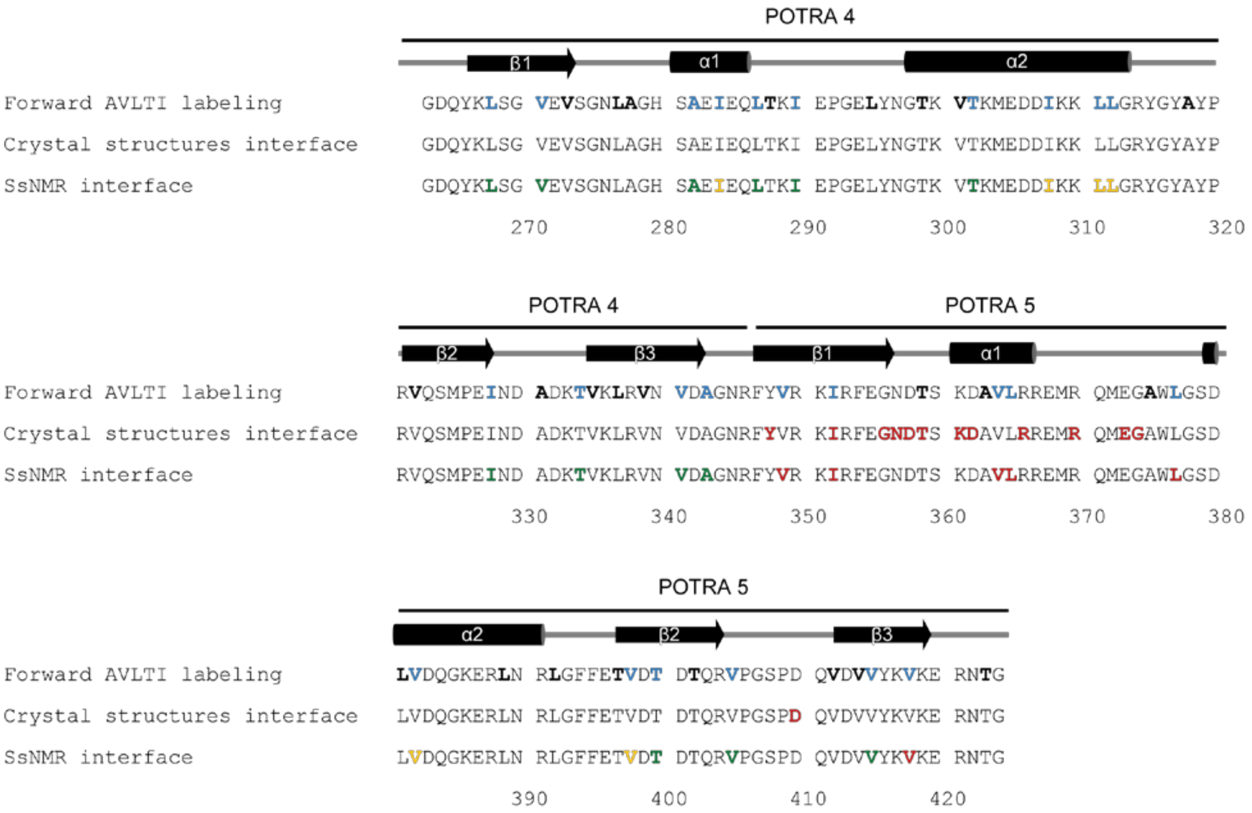

Supplementary Figure 2 BamCDE interaction interface plotted on the P4P5 sequence

Topology and secondary structural elements determined from solution NMR P4P5 assignments (BMRB 19928) using TALOS+[1] are depicted above the sequence for these domains. Upper sequence: Forward AVLTI labelling refers to specific ^{13}C and ^{15}N labelling of alanine, valine, leucine, threonine and isoleucine residues. In blue are the residues used in the analysis of complex formation as they have more than two isolated correlations (except for alanine) in the ^{13}C-^{13}C correlation spectra. In black and bold are those that are labelled but cannot be used in the analysis. Middle sequence: Crystal structures interface BamA-BamCDE interface as described by PDBePISA[2] on all Bam complex structures obtained by x-ray crystallography (PDB 5D0O, 5D0Q, 5AYW] and 5EKQ). In red, all residues on P4P5 that form contacts with residues from lipoproteins BamCDE. Lower sequence: ssNMR interface BamAP4P5-BamCDE interaction interface as described by ssNMR data. Residues in red correspond to those that show a chemical shift perturbation, yellow those that experience an increase in signal intensity and green those that do not experience any effect upon complex formation.

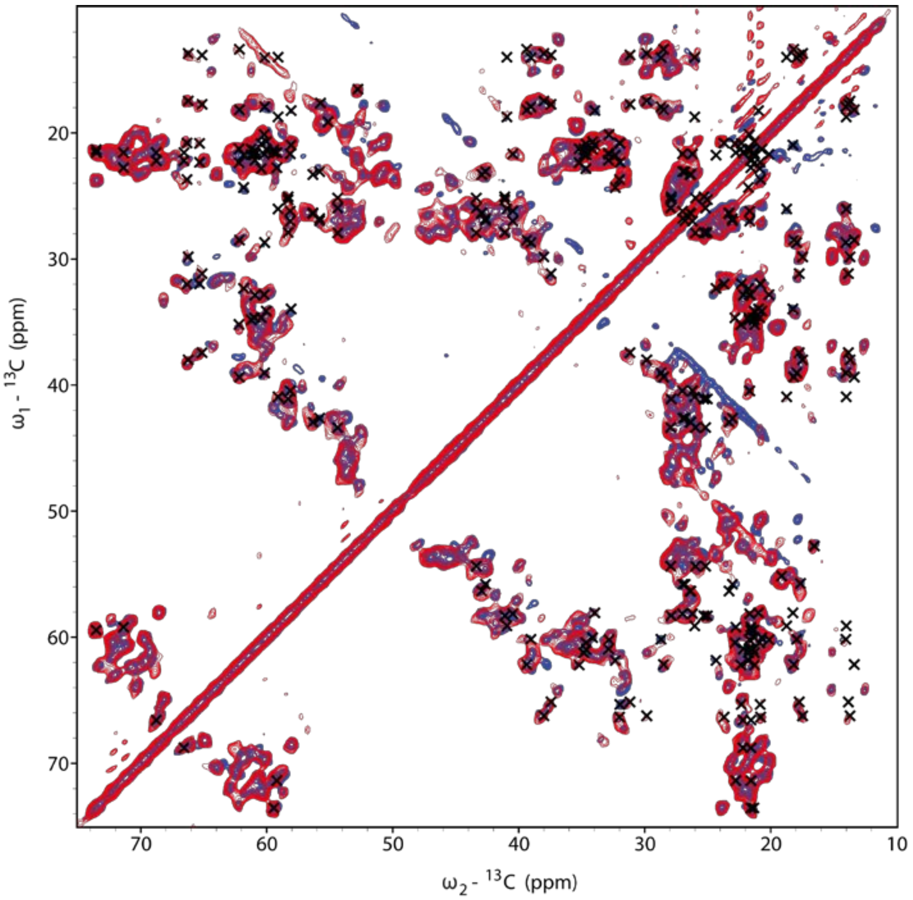

Supplementary Figure 3 ^{13}C detected ssNMR on the specifically AVLTI labelled BamAP4P5, in the absence and presence of the unlabeled BamCDE sub-complex

2D ^{13}C-^{13}C spectra of BamAP4P5 in liposomes, in the absence and presence of sub-complex BamCDE (blue and red spectra, respectively). Crosses are the NMR assignments of the AVLTI labelled residues assigned by the combination of solution and ^{1}H detection NMR experiments.

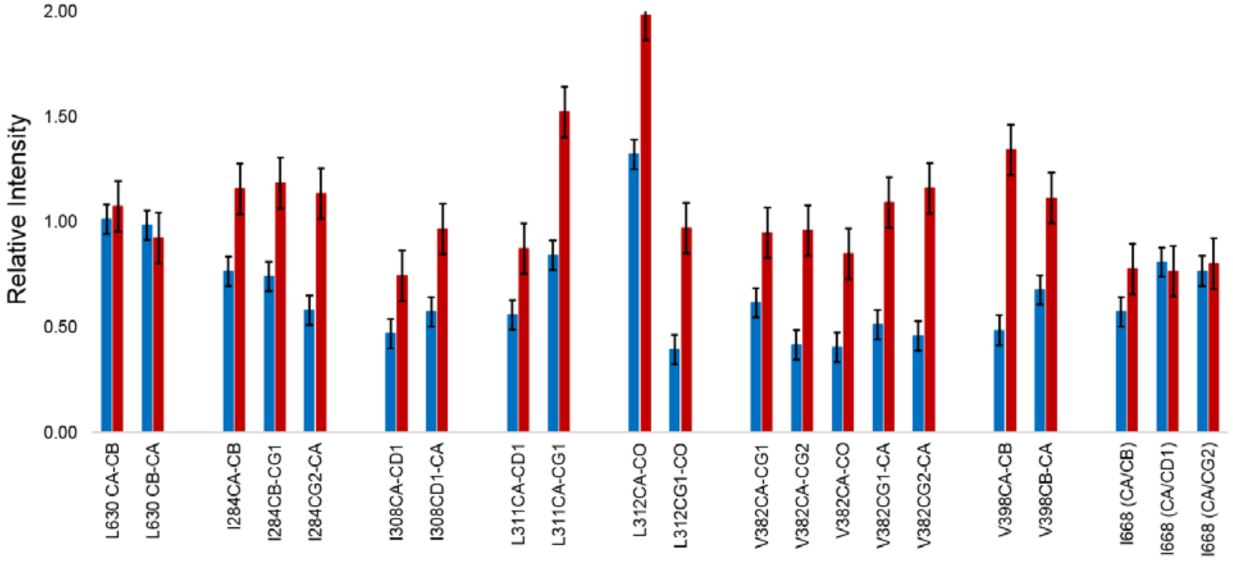

Supplementary Figure 4 Comparison of peak intensities for residues in BamAP4P5, relative to L630 of the TM domain

Relative intensities for several correlations of residues that exhibit alterations in peak intensities, in BamAP4P5 (blue bars) and in the presence of BamCDE (red bars). Also indicated are correlations for I668 of EL6. Error bars are derived from the noise in the respective spectra.

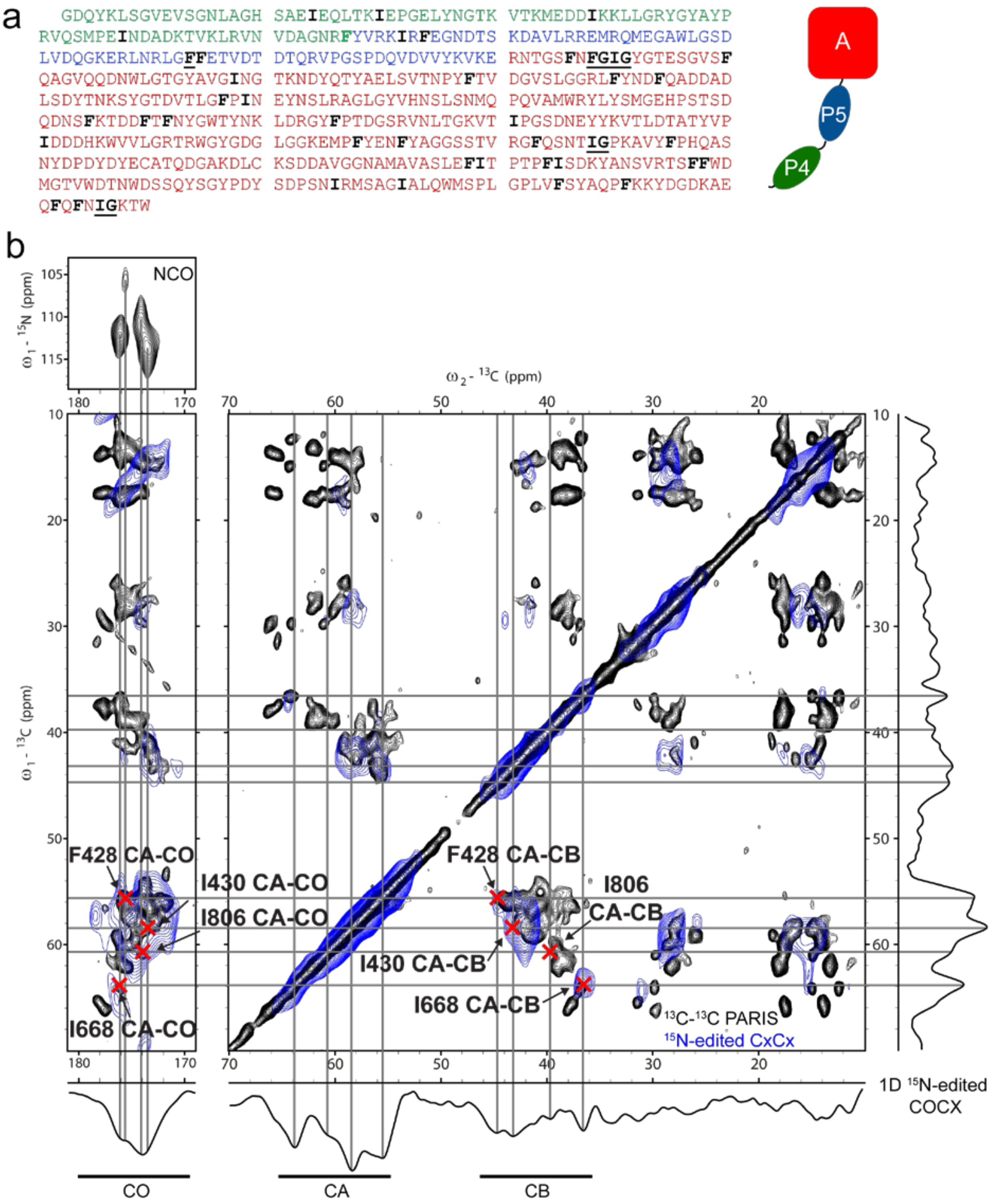

Supplementary Figure 5 Tentative assignment of specific IG and FG pairs within BamA TM

a) Sequence of BamAP4P5 (P4 in green, P5 in blue and TM β-barrel in red) where Ile and Phe residues are shown in black and underlined are the sequential IG and FG pairs and F394 discussed in the main text. **b)** Composite of recorded spectra for tentative assignment of the

specifically targeted IG and FG pairs. Spectra in black were recorded at set sample temperature of -2°C (700 MHz ^{1}H frequency), and the sample is not frozen as judged by the water peak (not shown). The ^{15}N-edited C_XC_X (30 ms mixing) spectrum in blue was recorded for the BamAP4P5 protein under DNP conditions (400 MHz ^{1}H frequency). Top left is the 2D NCO of the BamAP4P5 sample, the 1D ^{15}N-edited C_OC_X spectra surrounding the ^{13}C-^{13}C (30 ms mixing) PARIS spectrum which only targets the sequential pairs of interest, therefore limiting spectral overlap. Lines show the relationship between the correlations measured for the residues. Note that only Cα, Cβ and CO correlations are obtained for these residues. Combination of 1D ^{15}N-edited C_OC_X allowed for assignment of both F428 and I668 due to their characteristic chemical shifts (Cα characteristic for α-helical conformation of I668 and only Phe residue in a sequential pair for F428).Tentative assignments are given for I430 and I806, where I806 was assigned due to its lack of intensity both in 1D ^{15}N-edited C_OC_X and DNP 2D (blue) whereas I430 is more intense and follows F428 on β1.

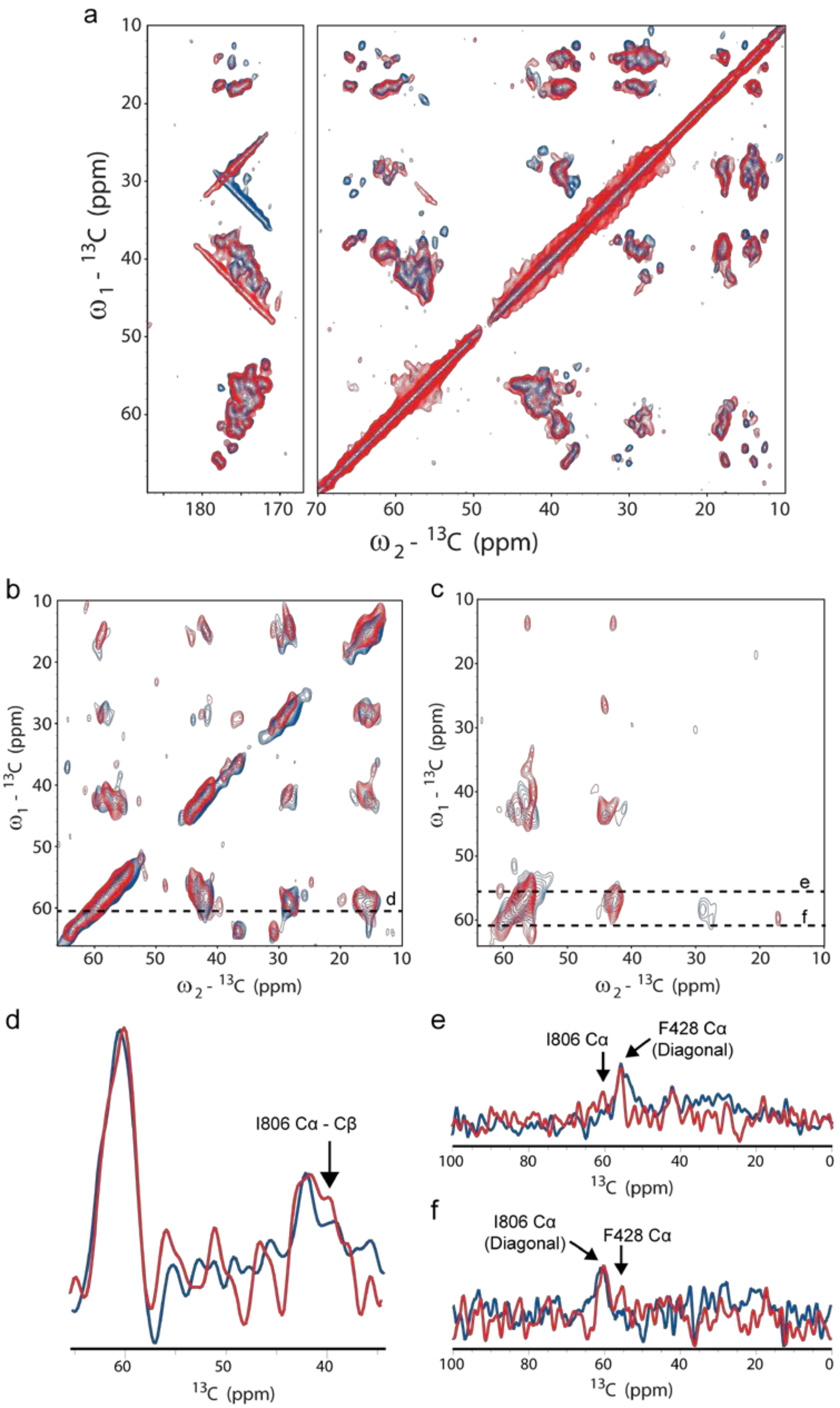
a
b
c
d
e
f
ω1 - 13C (ppm)
ω2 - 13C (ppm)
13C (ppm)
I806 Cα - Cβ
I806 Cα
F428 Cα
(Diagonal)
I806 Cα
(Diagonal)
F428 Cα

Supplementary Figure 6 ^{13}C detected ssNMR on the specifically ^{13}C-Ile/Phe, ^{15}N-Gly labelled BamAP4P5, in the absence and presence of the unlabeled BamCDE sub-complex

a) ^{13}C-^{13}C correlation spectra measured on a 700 MHz spectrometer at 258 K of the IGF labelled BamAP4P5 in the presence and absence of unlabeled BamCDE (blue and red spectra respectively). Crosses in green are the solution NMR assignments for the isoleucine and phenylalanine residues in the P4P5 domains. In black are the tentative assignments for the residues targeted by sequential experiments. **b and c)** ^{15}N-edited C_XC_X spectrum of the samples in A, measured on a 400 MHz DNP system at 100 K with 30 and 1000 ms mixing time respectively. Spectra are scaled on the I668 CA-CB correlation, from the EL6 loop. From the 2D spectra in b and c, 1D slices were extracted for cross sections at the indicated positions labeled **d, e and f.**

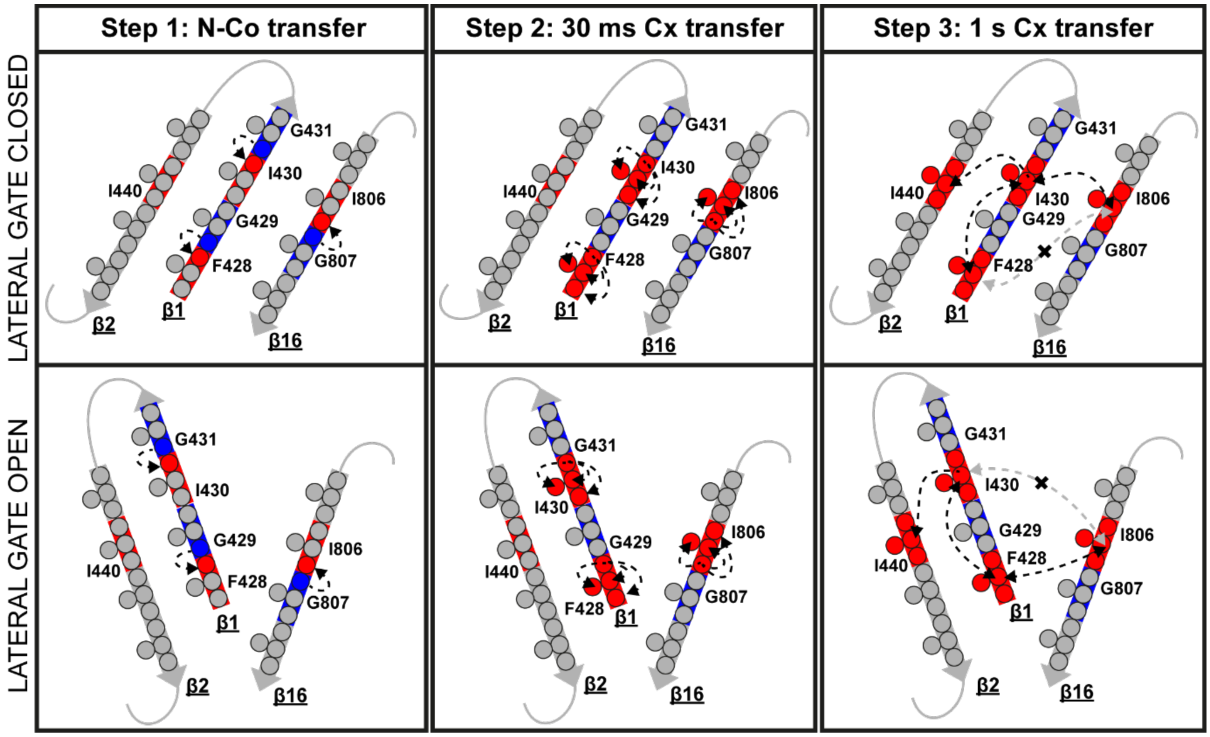

Supplementary Figure 7 Schematic diagram of ^{15}N-edited C_XC_X DNP experiments

Short (30 ms) and long (1s) mixing ^{15}N-edited C_XC_X experiments was performed identically for both BamA lateral gate open and closed situations. Residues within reach of magnetization transfer however vary. In gray are residues which are not labelled in the ^{13}C Ile/Phe and ^{15}N Gly labelling scheme. In blue are ^{15}N labelled glycines, red are ^{13}C Ile or Phe. When a circle is colored this indicates magnetization on this atom – magnetization transfer is indicated by the dashed lines. In step 1 magnetization is transferred specifically through N of glycine to Co of labelled Ile or Phe. In step 2 the magnetization is distributed through the residue. Step three is the 1 second mixing where magnetization is transferred to ^{13}C carbons in the vicinity of atoms

that have magnetization. Note the dashed arrow in grey that indicates the residues are too far away.

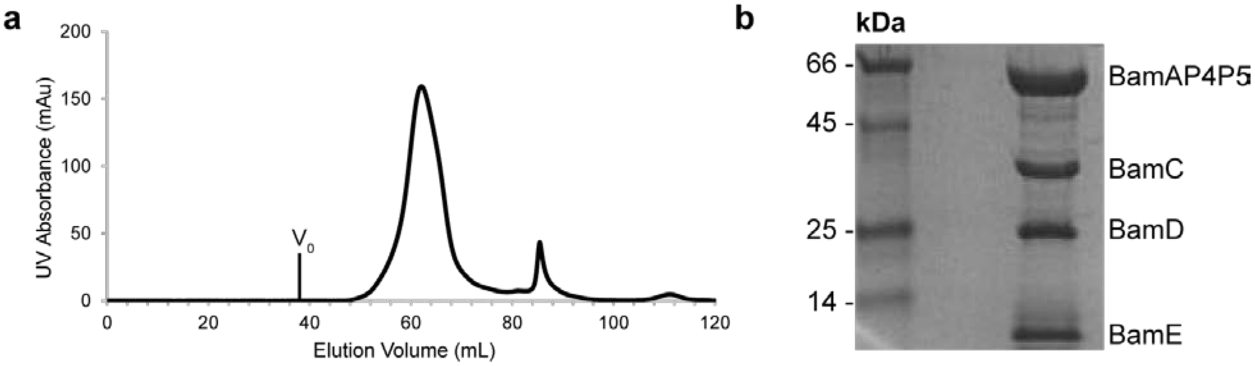

Supplementary Figure 8 BamAP4P5-BamCDE complex formation

a) Representative gel filtration chromatogram obtained on a Superdex 200 PG for a sample of detergent purified BamAP4P5-BamCDE. **b)** SDS-PAGE gel of a sample taken from the fraction at approximately 60 mL elution of the stable BamAP4P5-BamCDE complex.

Supplementary Tables

Supplementary Table 1 Chemical-shift assignments of P4P5 peaks assigned in Figure 2 as derived from the combination of published solution NMR assignments (BMRB 19928) and the NH, CaNH and CoNH PD spectra of the BamAP4P5 construct

Res. n°.	N	H	Cα	CO	Res. n°.	N	H	Cα	CO	Res. n°.	N	H	Cα	CO
267	120.55	8.24	55.46	176.40	310	120.97	8.612		178.36	364	115.59	7.13	64.83	
268	123.45	8.84	54.44	176.77	311	121.71	7.784		178.94	365	116.83	7.12	56.21	
269	119.40	9.36	57.98		312	115.99	8.031	57.792		368	114.40	7.17	54.41	176.03
270	106.45	7.04	45.02	170.55	313	107.79	8.286			369	112.59	7.03	54.92	
271	120.13	8.41	60.78		314	123.31	7.645	58.940		374	113.38	9.29		172.37
272	127.02	8.80			316	106.22	7.410		174.99	377	125.56	9.22		
275	106.47	8.23	43.81		317	122.75	7.628	57.293		382	123.46	8.07	66.03	
276	119.54	8.45	51.62		319	115.08	9.011	54.813		383	123.79	8.43	57.72	
280	116.51	7.75	55.03	175.11	327	124.37	9.146	55.403	175.90	384	121.19	8.76		179.99
281	114.83	7.84	61.94		328	129.55	8.800		175.32	385	104.27	7.70	46.75	174.76
282	123.59	8.59	55.21	181.10	330	123.40	8.729	56.606	177.28	386	121.93	7.58	59.77	
283	118.46	8.15	59.14	179.50	332	114.10	7.216	53.056	175.09	393	101.22	7.20		
284	119.61	7.87	64.74		334	108.49	8.050	59.207		394	114.02	5.90	54.88	
286	115.93	7.39	58.34	178.26	335	118.12	8.657	60.118		396	123.69	9.54	58.37	176.76
287	116.75	7.61	56.25	178.35	336	128.72	8.339	54.677	174.50	397	106.28	7.09	60.14	173.01
288	106.58	7.39	60.35	173.51	337	127.20	8.455	53.378	174.75	398	120.29	7.99	61.80	
289	120.33	6.74	56.71		338	125.80	8.013	54.917		400	109.65	8.45	58.93	
290	120.41	7.93	58.87		341	125.64	8.793	60.035		405	126.63	8.28	59.75	
297	123.42	7.20	53.17	175.08	343	127.26	8.435		176.74	408	113.94	7.83	53.36	
299	118.19	8.03	65.91	176.60	344	99.55	7.722	44.188		410	114.15	7.75	53.97	
300	122.26	7.63	58.22	179.17	346	121.37	7.976	55.125		411	114.61	7.50	54.00	
302	114.62	7.82	66.09	175.60	349	118.97	8.230	61.705	175.92	412	113.07	8.80	58.56	
304	118.35	7.83	58.87	178.93	350	131.35	9.684			415	126.03	8.53	59.48	175.76
305	116.47	8.13	60.00	178.90	351	114.18	7.920	53.419	174.92	416	125.13	8.69	56.71	
306	119.30	8.31		179.26	352	121.82	9.252	59.822		418	120.20	9.19		
307	121.37	8.93		180.11	356	110.84	8.803	44.003	174.05	419	119.85	7.46	54.07	
308	122.30	8.27	65.90		360	120.66	8.624	58.996		420	127.26	9.68		

Res. n°. refers to the residue number of the amino acid in the BamA sequence. Columns entitled: N, H, Cα and CO, refer to the chemical shifts associated with these nuclei for the corresponding residue.

Supplementary Table 2 ssNMR Analysis of dynamic (as seen in Solution NMR) POTRA residues

Residue	Proton detection	
	CαNH	CONH
A318	Not present[a]	Not present[a]
F347	Present	Crowded region
Y348	Crowded region[b]	NH crowded region, CO present
A363	Crowded region	Not present
V364	Present	Not present
R367	Crowded region	Not present
E368	Present	NH not present, CO present
G374	Weak	CO weak, NH not present
A375	Not present	Not present
L377	Present	NH weak
S379	No NH assignment from solution	
D383	Crowded region	NH Not present, CO weak
G385	Weak	NH present, CO weak
E396	Weak	Present, NH weak
T397	Weak	Co weak, NH crowded region
T400	Present	NH present, CO crowded region
T402	Crowded region	Crowded region
Q403	Present	Crowded region

[a)] Not present is either due to chemical shift differences between solution and ssNMR spectra or linewidths broadened beyond detection or within the noise of the spectrum. [b)] Crowded region could denote that the correlations are in spectrally complex region or an isolated peak that agrees in assignment but is then present in TM spectra.

Supplementary Table 3 Tentative assignments for residues identified in Supplementary Figure 3, at the lateral gate.

Residue	Cα	CB	CO
**F428**	55.68	44.69	176.00
**I430**	58.53	43.22	173.70
**I668**	64.18	36.74	176.10
**I806**	60.72	39.64	174.30

Supplementary Table 4 Parameters of ^{1}H ssNMR experiments in Figure 1

Sample	Spectrum	Temperature (K)	MAS (kHz)	Acquisition time (h)
BamAP4P5	NH	244	60	13.2
	CαNH	244	60	151.8
	CONH	244	60	89.3
BamATM	NH	244	59.5	13.2
	CαNH	244	59.5	197.1
	CONH	244	59.5	119.1

The 2D NH in Figure 1a, b and c were acquired for 15.0 ms and a spectral window of 61.0 ppm in t_1 and 29.8 ms and a spectral window of 10.0 ppm in t_2. The spectra were processed with a squared sine bell function 5 in t_1 and t_2, with 1024 and 4096 zero filling and no linear prediction. 3D CαNH in Fig. 1e, f and g were acquired for 5.0 ms in t_1 and 6.0 ms in t_2 with spectral window width of 28.0 ppm and 36.1 ppm in t_1 and t_2 respectively. The spectrum was processed using a squared sine bell function 3 in t_1 and t_2 and 4 in t_1, with zero filling of 128, 128 and 2048 in t_1, t_2, and t_3 respectively. 3D CONH in Fig. 1f was acquired for 5.5 ms in t_1 and 6.0 ms in t_2 with spectral width of 15.0 ppm and 36.1 ppm in t_1 and t_2 respectively. The spectrum was processed using a squared sine bell function 3 in t_1 and t_2 and 4 in t_1, with zero filling of 128, 128 and 2048 in t_1, t_2, and t_3 respectively. All experiments were conducted at 800MHz.

Supplementary Table 5 Parameters of 2D ^{13}C-^{13}C PARIS spectra of specifically labelled BamAP4P5 and BamAP4P5-BamCDE in Fig. 3 and 4 and Supplementary Fig. 3

Sample	Labelling	CP time (ms)	Mixing time (ms)	Temp (K)	MAS (kHz)	Acquisition time (h)
BamAP4P5	AVLTI	0.85	30	262	13	17.5
	IFG	0.8	30	258	13	20.6
BamAP4P5 + BamCDE	AVLTI	1.0	30	262	13	30.9
	IFG	0.9	30	258	13	82.5

The 2D ^{13}C-^{13}C PARIS data sets in Figure 3 were recorded with 5.9 ms in t_1 and 10.0 ms in t_2 with a spectral window of 140.0 ppm in t_1 and 338.1 ppm in t_2. The spectra were processed using squared sine bell function 4 in both t_1 and t_2, a zero filling of 2048 and 4096 was used in t_1 and t_2 respectively, with 4 linear prediction coefficients in t_1 and 2 linear prediction coefficients in t_2. The 2D ^{13}C-^{13}C PARIS data sets in Figure 4 and Supplementary Fig. 3 were recorded with 5.9 ms in t_1 and 10.0 ms in t2 with a spectral window of 140.0 ppm in t_1 and 338.1 ppm in t_2. The spectral were processed using a sine bell function 4 in both t_1 and t_2, a zero filling of 2048 was used in both t_1 and t_2, with no linear prediction. Data were recorded on 700 MHz NMR instrument.

Supplementary Table 6 Parameters of 2D ^{15}N-edited C_xC_x data in Figure 4 and Supplementary Figure 3 measured on the ^{13}C-Ile/Phe, ^{15}N-Gly BamAP4P5 on a 400 MHz ^{1}H machine with DNP setup

Sample	HN CP (μs)	SPECIFIC CP mixing (ms)	PDSD mixing (ms)	Temp (K)	MAS (kHz)	Acquisition time (h)
BamAP4P5	700	5	30	100	8	13.6
			1000			54.6
BamAP4P5 + BamCDE	700	5	30	100	8	13.6
			1000			54.6

The ^{15}N-edited C_xC_x experiments in Fig. 4 and Supplementary Fig. 3 were acquired for 3.0 ms in t_1 and 9.9 ms in t_2 with spectral width of 125.0 ppm and 405.4 ppm in t_1 and t_2 respectively. The spectra were processed using a squared sine bell function 2.5 in t_2 and t_1. In t_1 512 points of zero filling were used and in t_2 2048, with no linear prediction.

Supplementary references

1. Y. Shen *et al*., TALOS Plus: a Hybrid Method for Predicting Protein Backbone Torsion Angles From NMR Chemical Shifts, *J. Biomol. NMR* **44,** 213–23.

2. Protein interfaces, surfaces and assemblies service PISA at the European Bioinformatics Institute. (http://www.ebi.ac.uk/pdbe/prot_int/pistart.html) Evgeny Krissinel and Kim Henrick, “Inference of Macromolecular Assemblies From Crystalline State,” *J. Mol. Biol.* **372,** 774-797 (2007).
